# Expanding the Reach of an Evidence-Based, System-Level, Racial Equity Intervention: Translating ACCURE to the Maternal Healthcare and Education Systems

**DOI:** 10.3389/fpubh.2021.664709

**Published:** 2021-12-14

**Authors:** Stephanie L. Baker, Kristin Z. Black, Crystal E. Dixon, Christina M. Yongue, Hailey Nicole Mason, Patrick McCarter, Matthew Manning, Joanne Hessmiller, Ida Griesemer, Aditi Garikipati, Eugenia Eng, Daniel Kelvin Bullock, Claire Bosire, Kimberly M. Alexander, Alexandra F. Lightfoot

**Affiliations:** ^1^Greensboro Health Disparities Collaborative, Greensboro, NC, United States; ^2^Public Health Studies Department, Elon University, Elon, NC, United States; ^3^Department of Health Education and Promotion, East Carolina University, Greenville, NC, United States; ^4^Department of Public Health Education, University of North Carolina at Greensboro, Greensboro, NC, United States; ^5^Department of Health Behavior, University of North Carolina at Chapel Hill, Chapel Hill, NC, United States; ^6^Cone Health, Greensboro, NC, United States; ^7^Department of Social Work and Sociology (Retired), North Carolina Agricultural and Technical State University, Greensboro, NC, United States; ^8^Department of Cardiology, Duke University, Durham, NC, United States; ^9^Office of Equity Affairs, Durham Public Schools, Durham, NC, United States; ^10^The Alexander Group, Durham, NC, United States; ^11^Center for Health Promotion and Disease Prevention, University of North Carolina at Chapel Hill, Chapel Hill, NC, United States

**Keywords:** racism and antiracism, systems change in health care, community-based participatory research (CBPR), health disparities, intervention translation, racial equity, evidence base

## Abstract

The abundance of literature documenting the impact of racism on health disparities requires additional theoretical, statistical, and conceptual contributions to illustrate how anti-racist interventions can be an important strategy to reduce racial inequities and improve population health. Accountability for Cancer Care through Undoing Racism and Equity (ACCURE) was an NIH-funded intervention that utilized an antiracism lens and community-based participatory research (CBPR) approaches to address Black-White disparities in cancer treatment completion. ACCURE emphasized change at the institutional level of healthcare systems through two primary principles of antiracism organizing: transparency and accountability. ACCURE was successful in eliminating the treatment completion disparity and improved completion rates for breast and lung cancer for all participants in the study. The structural nature of the ACCURE intervention creates an opportunity for applications in other health outcomes, as well as within educational institutions that represent social determinants of health. We are focusing on the maternal healthcare and K-12 education systems in particular because of the dire racial inequities faced by pregnant people and school-aged children. In this article, we hypothesize cross-systems translation of a system-level intervention exploring how key characteristics of ACCURE can be implemented in different institutions. Using core elements of ACCURE (i.e., community partners, milestone tracker, navigator, champion, and racial equity training), we present a framework that extends ACCURE's approach to the maternal healthcare and K-12 school systems. This framework provides practical, evidence-based antiracism strategies that can be applied and evaluated in other systems to address widespread structural inequities.

## Introduction

Factors contributing to racial inequities in the United States (U.S.) are complex, influenced by historical and contemporary social injustices (namely racism), and range from the individual to system-level (1). Regardless of the system being examined, racial inequities in outcomes are pervasive due to structural, systemic, and institutional racism (hereafter referred to as racism) embedded in policies, practices, and cultures of these systems (2). Interventions that aim to reduce racial inequities have focused largely on individual-level factors, which have limited long-term effectiveness (3, 4) and do not address the system-level factors contributing to inequities. To effectively address racial inequities produced by racism, we must develop and implement multi-faceted, system-level interventions that address multiple levels of change (e.g., individual, interpersonal, community, institutional, and policy), and utilize best practices, including fully engaging community partners in this process and incorporating interventions into existing systems (3). This article describes the Accountability for Cancer Care through Undoing Racism and Equity (ACCURE) intervention and explores how key components could be applied to the maternal healthcare and education systems to address structural barriers to racial equity.

## Accountability for Cancer Care Through Undoing Racism^®^ and Equity

ACCURE was a longitudinal randomized control trial (RCT) funded by the National Cancer Institute (NCI) (Grant# 1R01CA150980-01A1) to reduce racial disparities in treatment completion between Black and White patients with stage I or II breast or lung cancer at two U.S. cancer centers (5). The study implemented a multi-faceted, quality improvement intervention to address structural barriers to care that were identified in formative studies (6) and informed by a community-based participatory research (CBPR) approach (7). Evidence from routine clinical practice was used to improve quality of cancer care, which resulted in improved treatment completion for both Black and White patients, and the elimination of disparity between the two racial groups (5).

**Figure 1:**
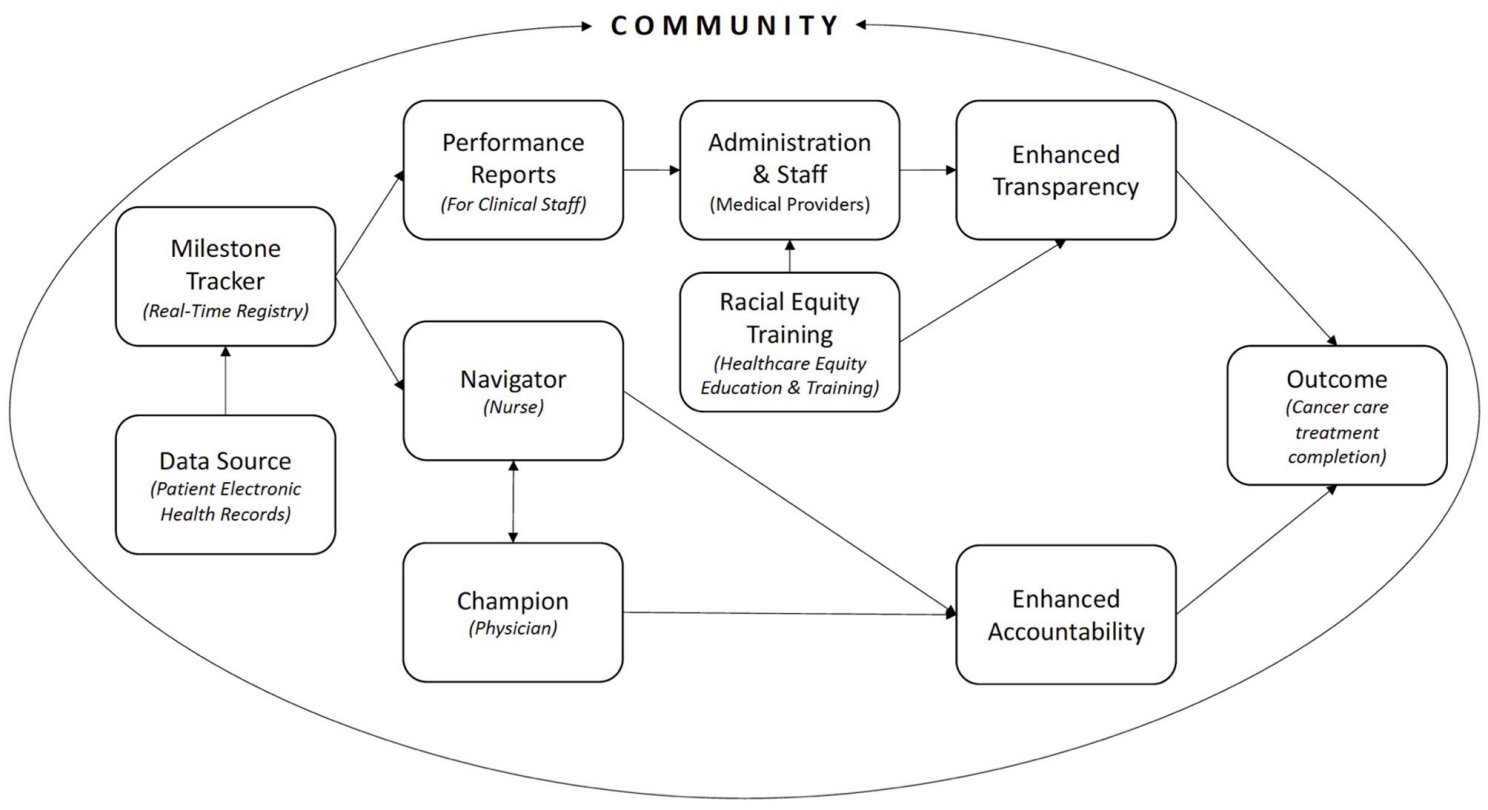
ACCURE component: from real-world data to enhanced transparency and accountability.

The Greensboro Health Disparities Collaborative (GHDC) is a community-medical-academic partnership working toward racial equity in healthcare (8, 9). Please see the Supplementary Materials for a deeper description of the partnership. GHDC developed ACCURE, an intervention unique in its application of CBPR, key antiracism principles of transparency and accountability, and system-level change, which can be translated to other systems to reduce racial inequities and improve overall outcomes. Figure 1 depicts the key intervention components of ACCURE and how the use of electronic health record (EHR) data in combination with the real-time registry (RTR), nurse navigator, physician champion, clinical performance reports, and Healthcare Equity Education and Training (HEET) sessions led to enhanced transparency and accountability in the cancer care system (7). This figure is encapsulated within the community to signify the critical and necessary community-centered nature of ACCURE and how community members were involved at every step. Details of ACCURE are available in previous publications (9) and brief descriptions of the core components are below.

### Community Partners

GHDC consists of community members, healthcare providers, and academic researchers from central North Carolina who have either been a survivor of the diseases we are studying, self-identify as a member of an underrepresented population group (by race, gender, age, and/or religious faiths), or their professional or personal work focuses on understanding and eliminating racial inequities. Each GHDC member is required to attend the Undoing Racism^®^ Training (10) or the Racial Equity Institute (REI) Phase 1 training (11). The GHDC maintained power to review and approve deviations from ACCURE's plan, progress reports to NCI, and all dissemination products. GHDC members served on every committee involved in the ideation, formation, and design of the intervention, as well as the implementation, dissemination, and evaluation. Community member input was critical to ACCURE's success.

In ACCURE, The Partnership Project, Inc. (TPP) (12), a Greensboro, NC based non-profit antiracism training organization, served as the community partner organization. The Executive Director of TPP was designated in the grant contract as the official community partner site director. GHDC members and TPP's Board of Directors ensured that research methods and findings remained relevant to community needs.

### Outcome

Eliminating racism in healthcare has long-lasting benefits on patient treatment and survival. Yet, it is critical to identify proximal outcomes with a direct impact on long-term results. The proximal outcome can serve as a leading metric since equity is a lagging metric. In oncology, completion of recommended treatment correlates with full recovery and was the outcome of interest (5).

In ACCURE, (5) there was a statistically significant racial disparity in the retrospective group's treatment completion rates (79.8% for Black patients, 87.3% for White patients, *p* < 0.001). In the intervention group, this racial gap not only disappeared, but the treatment completion rates improved for both Black and White patients (88.4 and 89.5%, *p* = 0.77).

### Milestone Tracker

The real-time registry (RTR), derived from patients' EHR data, was developed for ACCURE to track patient milestones by race. This database was updated daily to track anticipated milestones in each patient's cancer treatment journey. The RTR alerted the navigator and physician champion (both roles described below) about missed appointments and unmet milestones [i.e., standards of care; (5)].

### Navigator

Navigators assist patients through the complex levels of institutional procedures (e.g., setting appointments, connections with organizational resources, providing health education, and/or advocating for patient's concerns to providers). ACCURE Navigators were nurses (with minimally a Bachelor of Science in Nursing and management experience) who went through additional training in antiracism analysis and patient-centered care protocols [i.e., the Teach Back method, Kleinman's Patient Model, and instructions on using the RTR; (13)].

ACCURE Navigators assisted cancer patients from diagnosis through their active treatment completion journey. Whereas, usual-care navigators serve as advocates for providers (a one-way link) to ensure patient compliance/adherence to medical regimen, ACCURE Navigators served as patient advocates and a two-way liaison between patients and providers.

### Champion

In ACCURE, this role was filled by established powerholders within the institution - cancer care physicians. They advocated for and promoted the intervention, as well as directly supported the ACCURE Navigator. They communicated study updates and reports regarding individual performance to their cancer care teams using institutional language and relationships. Ultimately, the Champions lend credibility to the project among institutional peer partners.

### Racial Equity Training

Concepts from REI's antiracism trainings were adapted into healthcare system-specific focused sessions, delivered quarterly for cancer center providers and staff. These Healthcare Equity Education and Training (HEET) sessions covered antiracism topics (e.g., transparency, accountability, and gatekeeping) and presented site-specific Clinical Performance Reports on patient outcomes by race (13). This component allowed for transparent discussions among multidisciplinary institutional members, so that further solutions and action could be implemented to address systemic racism within their institutions.

### Purpose of This Article

“Real-world data,” (RWD) an umbrella term used to describe the benefit, risk, and resource use of health data that are not collected through conventional RCTs (14), are increasingly used to make decisions in healthcare because they capture nuances in patient journeys (15). RWD can be generated from routine clinical practice observations or collected retrospectively from a variety of sources such as EHR data, claims/billing activities, and patient-reported outcomes (14).

**Figure 2:**
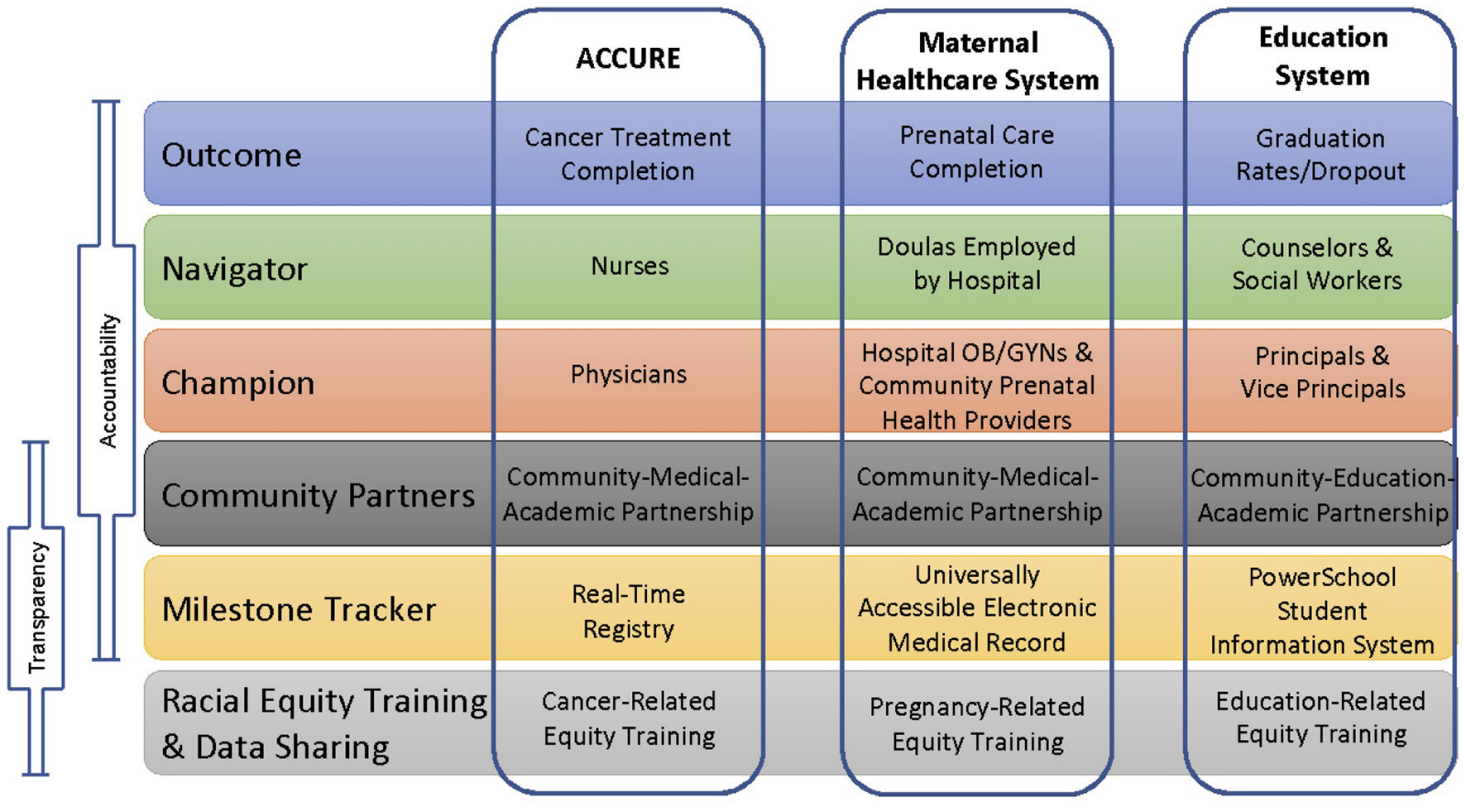
Core components of ACCURE and representative examples in the maternal healthcare and education systems.

ACCURE utilized RWD to eliminate racial disparities in cancer care (Figure 1) and can be applied beyond healthcare to address the antiracism principles of transparency and accountability in other systems. The maternal healthcare and education systems are two systems where People of Color can have widely varying experiences and outcomes compared to their White counterparts. In this article, we propose translating the key components of the ACCURE intervention into the maternal healthcare and education systems to address racism. Figure 2 shows the core components of ACCURE with the related applications to maternal healthcare and education described in greater detail below.

## Translation to the Maternal Healthcare System

Racial inequities in maternal health outcomes are well-documented. Black women are three times more likely to experience maternal mortality than White women and have worse incidences of severe maternal morbidity (16, 17). College educated Black women have pregnancy-related mortality rates that are worse than White women who have not completed high school (17). The racism that persists in our maternal healthcare system contributes to poor maternal health outcomes among women of all races and ethnicities in the U.S. compared to our peer countries (18), demonstrating the importance of translating ACCURE to the maternal healthcare system.

Addressing racial inequities in maternal healthcare can improve outcomes for all pregnant people in a similar way that ACCURE eliminated Black-White racial gaps in treatment completion and improved cancer care for all patients in the study. While the maternal healthcare system has some similarities to the cancer care system, there are appreciable differences.

### Community Partners

Similar to ACCURE, a community-medical-academic partnership that includes people who have experienced pregnancy and their partners, maternal healthcare providers [e.g., doulas, midwives, nurses, obstetrician/gynecologists (OB/GYNs), and administrative staff], community organizers, and academic researchers with CBPR and racial equity expertise will be crucial for success. Champions, described below, should be members of the partnership as well.

### Outcome

An important maternal healthcare outcome is reducing racial disparities in completion of prenatal care visits among pregnant people with hypertension. While prenatal care visits are important for monitoring health and well-being, these visits are vital for people at increased risk of pregnancy complications due to hypertension or preeclampsia which occurs in 1 in every 12–17 pregnancies (19, 20). Hypertension complicates pregnancy by increasing the likelihood of seizures, strokes, maternal mortality, and other ailments during delivery (21). Black women experience pregnancy- related death due to preeclampsia at 3.1 times the rate of White women (19) and risk for preeclampsia does not lessen for Black women with higher SES (22). If detected early, caregivers can facilitate the reduction of these risks by prescribing treatments to lower a patient's blood pressure or discussing lifestyle changes to help regulate stress (21). Prenatal care visits provide caregivers with regimented schedules to assess and monitor the health of pregnant people and their developing babies, to obtain accurate medical histories and prepare in advance for potential delivery complications (23).

### Milestone Tracker

We recommend the use of EHR data that contains relevant information about social demographics, health, and prenatal care of pregnant people and their developing babies, and records of patients' receipt of universally accepted standards of care for hypertension during pregnancy (5). This real-time data source must be housed and programmed at the hospital and accessible to both prenatal care providers (e.g., primary care and reproductive healthcare providers) and hospital OB/GYN providers for data entry and monitoring. The universally accessible EHR will help to improve health outcomes for pregnant people and babies by (1) measuring the rate at which patients complete the full regimen of prenatal care visits, (2) ensuring transmission of relevant health information (e.g., blood pressure history and stress levels) between prenatal care providers and regional delivery centers, and (3) communicating alerts of missed prenatal care appointments or procedures. RWD must report health outcomes and missed appointments and procedures by race to increase transparency about racial inequities in maternal healthcare.

### Navigator

A navigator identifies and intervenes on structural issues complicating the patients' use of prenatal care and serves as a two-way liaison between patients and care providers during prenatal, perinatal, and postpartum time periods. A doula on staff at the hospital could function as navigator and must be trained in antiracism principles and using the milestone tracker. Having access to the EHR milestone tracker allows the navigator to identify racial inequities in outcomes in real-time which can increase accountability by affording the maternal healthcare system an opportunity to efficiently tackle structural barriers contributing to the inequities. Access to the milestone tracker is critical for the champion as well.

### Champion

In the case of maternal healthcare where people may have multiple providers both within and outside of the hospital, multiple champions will be necessary. Within the hospital system, the champion should be an OB/GYN with leadership responsibilities. Within the primary care system, the champion should be a physician, nurse midwife, or physician assistant, who has a leadership role and network connections to other providers in the community. Some local health departments provide access to prenatal care as well, requiring an additional champion identified within the health department. Champions, in addition to navigators, must monitor the EHR milestone tracker to identify and act on racial inequities that arise in real-time.

### Racial Equity Training

The historical and contextual foundation of racial disparities in maternal health outcomes is significant and substantive. Racial Equity Training that enables understanding of the structural and systemic origins of racial inequities (i.e., racism) in maternal and child health must be created to provide hospital and clinic employees with knowledge of the problem through a racial equity lens. This training must include a presentation of monthly reports, disaggregated by race, on prenatal visit completion and relevant clinical measures, which would mirror the race-specific transparency data or “Clinical Performance Reports” in ACCURE.

## Translation to the Education System

As a social determinant of health, education affects social and economic opportunities, physical health and well-being, and life trajectories. School dropout is associated with worse self-reported health and use of illicit drugs (24), asthma, diabetes, and heart disease (25). Education is regarded as the great equalizer, yet the racial gap in academic outcomes is vast. Translating ACCURE to this system may address these inequities.

### Community Partners

Schools are often closely connected to coalitions of community stakeholders. These coalitions are essential to holding the institution accountable for equitable practices and can include advisory councils, affinity groups, parent councils, students, and residents. Community partners should ensure that racial equity practices that create accountability are adopted and sustained in the school processes. Creating transparency with administrators, teachers, and community members is essential to building accountability for equity.

### Outcome

Since there are no comprehensive national standards for graduation requirements and states set them individually, ACCURE's proximal outcome cannot be perfectly translated to the education system. Graduation and dropout rates could be substitutes, though they are not an exact match to ACCURE's outcome. The data show inequities in graduation and dropout rates by race. White, Black, and Hispanic/Latinx students' dropout rates are 4.2, 6.4, and 8.0%, respectively (26). In the case of high school graduation, reports from the National Center for Education Statistics show that the adjusted cohort graduation rate for White students is higher than that of Black, Indigenous, and Hispanic/Latinx students by 10, 15, and 8%, respectively (26). These numbers demonstrate the need for new approaches to meet the needs of students of color in the academic environment.

### Milestone Tracker

Using North Carolina as an example, PowerSchool, a real-time data tracking tool, is used in public and private schools to manage student data and school processes and could serve as a milestone tracker. This tool gives parents, students, teachers, and administrators access to real-time data such as students' grades, attendance, and assignments. An important variable to track that is a leading indicator for graduation or dropout is truancy (i.e., excessive, repeated, and/or unexcused absences), which may negatively affect a child's ability to learn, grow, and graduate (27). According to the U.S. Department of Education, White, Black, and Hispanic absenteeism rates are 14.5, 20.5, and 17.0%, respectively (28). Black students are 40% more likely to lose 3 weeks of school compared to their White counterparts (28). The NC Department of Instruction suggests chronic absenteeism is an effective and actionable measure to serve as an early warning indicator to identify students with high truancy (27) and using this RTR could be an important tool to increase transparency about racial inequities. Strategies to reduce absences and truancy must not simply address systemic issues with individualized approaches, such as individualized plans for students, as the education system has historically done. Such strategies do not address root causes of racial inequities and are not effective. Highlighting system-level problems, such as differences in absenteeism across race, will lay the groundwork for new and system-focused solutions which can increase transparency and accountability in systems. Importantly, solutions must not involve police or additional punishment as this may increase long-lasting racial inequities in youth incarceration and justice involvement.

### Navigator

In the education system, school counselors and social workers (SCSWs) support students in completing their education. SCSWs are the frontline for student support, providing social, and emotional support *via* relationship-building with students, and instrumental support to help them navigate the system. They can also make suggestions for how systems can work more effectively for students. SCSWs as navigators must be trained in antiracism principles and in how to use the real-time milestone tracker PowerSchool. SCSWs must then help students navigate the complex structure of the education system while encouraging two-way communication between school personnel and students and their families.

### Champion

Within schools, school leadership, such as the principals and assistant principals, should serve as champions. Principals and assistant principals would receive notifications from PowerSchool about absences and truancy and share these data with teachers and school leaders. Ideally, any administrator at each individual or group of schools who has the power to enforce racial equity- centered interventions and ensure that all students receive equitable opportunities to succeed, should serve as champions. These leaders could set the tone for equity by ensuring race-specific data are collected, monitored, and used as the basis of decision-making. School administrator champions should work closely with and support the efforts of SCSW navigators.

### Racial Equity Training

School districts that provide professional development to teachers and administrators on a regular basis should include education-specific racial equity training. These trainings would need to include a historical and contextual analysis of race and racism in education, perhaps building on current tools, such as Root Cause Analysis, which helps teachers delve into the underlying and systemic causes of suspension rate disparities to prevent teacher bias from explaining away these disparities (29). Absence and truancy data, disaggregated by race, should also be shared during these trainings. Resources must be put in place to build an equitable school environment. The application of the ACCURE components to the education system provides an opportunity to think through how education systems can be accountable for decreasing racial disparities in graduation rates.

## Discussion

Racism is prevalent and pervasive in all systems. Recent global events, such as the coronavirus pandemic, have made the public more aware of racism and its impact on the lived experiences of minoritized populations and low-income communities. Yet, there is ample opportunity to use decades of lessons learned from racial equity scholars and community organizers in a variety of fields to assist with measuring and intervening on racism.

The success of ACCURE exemplifies how implementing system-level changes can ultimately lead to elimination of racial disparities in cancer care. In this article, we have described core components of ACCURE and proposed how these components can be translated in the maternal healthcare and K-12 education systems. We have noted key people that should be involved in and lead efforts to monitor RWD and implement equitable policies and practices that reverse past racial inequities produced by these systems. Above all, we stress the importance of the communities most impacted by these issues serving as core partners and leaders in every step of the process. Those who are most proximate to problems within systems must be equipped with the institutional knowledge, resources, and tools to translate ACCURE to their systems. In order to achieve equitable outcomes, each system must have transparent conversations about how racial inequities show up in their institutions. This can take the form of institutions analyzing their own data by race, as well as educating employees and communities being served about systemic racism, so that effective, accountable solutions can be developed to eliminate inequities.

Achieving racial equity will not happen overnight. Although eliminating racism and preventing racial inequities are our ultimate, long-term goals, it is imperative that we monitor leading, proximal outcomes (e.g., cancer treatment completion, prenatal care completion, and absenteeism/truancy) by race to identify where racial inequities are appearing. This continuous surveillance of short-term outcomes will allow us to intervene on racial inequities before they become severe or even fatal. We hope that the lessons learned from ACCURE will be translated to other systems to intervene in innovative ways against racism.

## Data Availability Statement

The original contributions presented in the study are included in the article/Supplementary Material, further inquiries can be directed to the corresponding author.

## Ethics Statement

The studies involving human participants were reviewed and approved by University of North Carolina Institutional Review Board. The patients/participants provided their written informed consent to participate in this study.

## Author Contributions

SB led the writing team through the conceptualization, development, and writing of the manuscript, was responsible for streamlining edits, recommendations and final submission, and also led the Maternal Healthcare section. KB and CD were part of the leadership team and met with SB to provide additional guidance. KB led the Introduction and CD led the Education System section. SB, KB, CD, CY, HM, PM, MM, JH, IG, AG, EE, DB, CB, KA, and AL contributed to the conceptualization, writing, editing, feedback, and approval of the final manuscript. All authors contributed to the article and approved the submitted version.

## Conflict of Interest

The authors declare that the research was conducted in the absence of any commercial or financial relationships that could be construed as a potential conflict of interest.

## Publisher's Note

All claims expressed in this article are solely those of the authors and do not necessarily represent those of their affiliated organizations, or those of the publisher, the editors and the reviewers. Any product that may be evaluated in this article, or claim that may be made by its manufacturer, is not guaranteed or endorsed by the publisher.

## References

[1] Purnell TS Calhoun EA Golden SH Halladay JR Krok-Schoen JL Appelhans BM. Achieving health equity: closing the gaps in health care disparities, interventions, and research. Health Affairs. (2016) 35:1410–5. 10.1377/hlthaff.2016.0158 27503965

[2] Jones CP. Levels of racism: a theoretic framework and a gardener's tale. Am J Public Health. (2000) 90:1212–5. 10.2105/AJPH.90.8.1212 10936998 PMC1446334

[3] Chin MH Clarke AR Nocon RS Casey AA Goddu AP Keesecker NM. A roadmap and best practices for organizations to reduce racial and ethnic disparities in health care. J Gen Intern Med. (2012) 27:992–1000. 10.1007/s11606-012-2082-9 22798211 PMC3403142

[4] Clarke AR Goddu AP Nocon RS Stock NW Chyr LC Akuoko JAS. Thirty years of disparities intervention research: what are we doing to close racial and ethnic gaps in health care? Med Care. (2013) 51:1–14. 10.1097/MLR.0b013e3182a97ba3 24128746 PMC3826431

[5] Cykert S Eng E Manning MA Robertson LB Heron DE Jones NS. A multi-faceted intervention aimed at Black-White disparities in the treatment of early stage cancers: the ACCURE pragmatic quality improvement trial. J Natl Med Assoc. (2020) 112:468–77. 10.1016/j.jnma.2019.03.001 30928088

[6] Black KZ Lightfoot AF Schaal JC Mouw MS Yongue C Samuel CA. *‘It’s like you don't have a roadmap really'*: using an antiracism framework to analyze patients' encounters in the cancer system. Ethn Health. (2018) 26:676–96. 10.1080/13557858.2018.1557114 30543116 PMC6565499

[7] Schaal JC Lightfoot AF Black KZ Stein K White SB Cothern C. Community- guided focus group analysis to examine cancer disparities. Prog Commun Health Partnersh. (2016) 10:159–67. 10.1353/cpr.2016.0013 27018365 PMC4810449

[8] Yonas MA Jones N Eng E Vines AI Aronson R Griffith DM. The art and science of integrating Undoing Racism with CBPR: challenges of pursuing NIH funding to investigate cancer care and racial equity. J Urban Health. (2006) 83:1004–12. 10.1007/s11524-006-9114-x 17072760 PMC3261297

[9] Eng E Schaal JC Baker SL Black K Cykert S Jones NS. Partnership, transparency, and accountability: changing systems to enhance racial equity in cancer care and outcomes. In: Wallerstein N Duran B Oetzel JG Minkler M, editors. Community-Based Participatory Research for Health: Advancing Social and Health Equity. San Francisco, CA: Jossey-Bass (2018). p. 107–22.

[10] The People's Institute for Survival and Beyond. Available online at: http://www.pisab.org/ (accessed January 27, 2021).

[11] Racial Equity Institute. Available online at: https://www.racialequityinstitute.com/ (accessed April 10, 2019).

[12] The Partnership Project. The Partnership Project, Inc. Available online at: http://thepartnershipproject.org/ (accessed January 27, 2021).

[13] Black KZ Baker SL Robertson LB Lightfoot AF Alexander-Bratcher KM Befus D. Health care: antiracism organizing for culture and institutional change in cancer care. In: Racism: Science & Tools for the Public Health Professional. Washington, DC: American Public Health Association (2019). p. 283–313. 10.2105/9780875533049ch14

[14] Makady A Boer AD Hillege H Klungel O Goettsch W. What is real-world data? A review of definitions based on literature and stakeholder interviews. Value in Health. (2017) 20:858–65. 10.1016/j.jval.2017.03.008 28712614

[15] Lipworth W. Real-world data to generate evidence about healthcare interventions. ABR. (2019) 11:289–98. 10.1007/s41649-019-00095-1 33717317 PMC7747250

[16] Admon L Winkelman T Zivin K Terplan M Mhyre J Dalton V. Racial and ethnic disparities in the incidence of severe maternal morbidity in the United States, 2012–2015. Obstet Gynecol. (2018) 132: 1158–66. 10.1097/AOG.0000000000002937 30303912

[17] Petersen EE Davis NL Goodman D Cox S Syverson C Seed K. Racial/ethnic disparities in pregnancy-related deaths—United States, 2007–2016. MMWR Morb Mortal Wkly Rep. (2019) 68:762–5. 10.15585/mmwr.mm6835a3 31487273 PMC6730892

[18] OECD. Health Status: Maternal and Infant Mortality. (2018). Available online at: https://stats.oecd.org/index.aspx?queryid=30116 (accessed December 15, 2020).

[19] MacKay AP Berg CJ Atrash HK. Pregnancy-related mortality from preeclampsia and eclampsia. Obstet Gynecol. (2001) 97:533–8. 10.1097/00006250-200104000-00011 11275024

[20] CDC. High Blood Pressure during Pregnancy. Centers for Disease Control and Prevention (2020). Available online at: https://www.cdc.gov/bloodpressure/pregnancy.htm (accessed January 11, 2021).

[21] Steegers EA von Dadelszen P Duvekot JJ Pijnenborg R. Pre-eclampsia. Lancet. (2010) 376:631–44. 10.1016/S0140-6736(10)60279-6 20598363

[22] Ross KM Dunkel Schetter C McLemore MR Chambers BD Paynter RA Baer R. Socioeconomic status, preeclampsia risk and gestational length in Black and White women. J Racial Ethn Health Disparities. (2019) 6:1182–91. 10.1007/s40615-019-00619-3 31368002

[23] Alexander GR Kotelchuck M. Assessing the role and effectiveness of prenatal care: History, challenges, and directions for future research. Public Health Rep. (2001) 116:306–16. 10.1016/S0033-3549(04)50052-3 12037259 PMC1497343

[24] Lansford JE Dodge KA Pettit GS Bates JE. A public health perspective on school dropout and adult outcomes: a prospective study of risk and protective factors from age 5 to 27 years. J Adolesc Health. (2016) 58:652–8. 10.1016/j.jadohealth.2016.01.014 27009741 PMC4877222

[25] The NCES Fast Facts Tool Provides Quick Answers to Many Education Questions. National Center for Education Statistics. Available online at: https://nces.ed.gov/fastfacts/display.asp?id=16 (accessed January 27, 2021).

[26] Vaughn MG Salas-Wright CP Maynard BR. Dropping out of school and chronic disease in the United States. J Public Health. (2014) 22:265–70. 10.1007/s10389-014-0615-x 25232516 PMC4164164

[27] NC DPS: Juvenile Justice Truancy Teams Work for NC Families. Available online at: https://www.ncdps.gov/blog/2017/05/17/juvenile-justice-truancy- teams-work-nc-families (accessed January 27, 2021).

[28] U.S. Department of Education. Chronic Absenteeism in the Nation's Schools. Available online: https://www2.ed.gov/datastory/chronicabsenteeism.html (accessed January 27, 2021).

[29] Purposes of Root Cause Analysis in School Improvement Planning. Office of Elementary and Secondary Education. Available online at: https://oese.ed.gov/resources/oese-technical-assistance-centers/state-support-network/resources/purposes-root-cause-analysis-school-improvement-planning (accessed January 27, 2021).

