# Supplementary material for: Expanding the Reach of an Evidence-Based, System-Level, Racial Equity Intervention: Translating ACCURE to the Maternal Healthcare and Education Systems
Source: Front Public Health. 2021 Dec 14;9:664709. doi: 10.3389/fpubh.2021.664709 (PMC8712314; doi:10.3389/fpubh.2021.664709)
Supplement: Supplementary file 1 [file Data_Sheet_1.docx]

Supplementary Material

# Supplementary Data

Additional details about the Greensboro Health Disparities Collaborative community-based participatory research partnership, including our history, members, and a list of our publications, can be found by visiting our website: <http://greensborohealth.org/index.html>.
